# CO_2_ and fire influence tropical ecosystem stability in response to climate change

**DOI:** 10.1038/srep29587

**Published:** 2016-07-18

**Authors:** Timothy M. Shanahan, Konrad A. Hughen, Nicholas P. McKay, Jonathan T. Overpeck, Christopher A. Scholz, William D. Gosling, Charlotte S. Miller, John A. Peck, John W. King, Clifford W. Heil

**Affiliations:** 1 Department of Geosciences, The University of Texas at Austin, 2305 Speedway, Stop C1160, Austin, TX 78712 USA; 2 Department of Marine Chemistry and Geochemistry, Woods Hole Oceanographic Institution, 360 Woods Hole Rd, Woods Hole, MA 02543 USA; 3 School of Earth Sciences and Environmental Sustainability, Northern Arizona University, Box 4099, Flagstaff, AZ 86011 USA; 4 Institute for the Environment, University of Arizona, ENR2 Building, 1064 E. Lowell St., Tucson, AZ 85721 USA; 5 Department of Earth Sciences, Syracuse University, Heroy Geology Laboratory, Syracuse, NY 13244-1070 USA; 6 Institute of Biodiversity & Ecosystem Dynamics, University of Amsterdam, POSTBUS 94248, 1090 GE Amsterdam, The Netherlands; 7 Department of Environment, Earth and Ecosystems, The Open University, Walton Hall, Milton Keynes, MK7 6AA, United Kingdom; 8 Department of Geosciences, University of Oslo, PO Box 1047, Blindern, Oslo 0316, Norway; 9 Department of Geosciences, University of Akron, 126 Crouse Hall, Akron, OH 44325-4101 USA; 10 Graduate School of Oceanography, University of Rhode Island, 215 South Ferry Road, Narragansett, RI 02882 USA

## Abstract

Interactions between climate, fire and CO_2_ are believed to play a crucial role in controlling the distributions of tropical woodlands and savannas, but our understanding of these processes is limited by the paucity of data from undisturbed tropical ecosystems. Here we use a 28,000-year integrated record of vegetation, climate and fire from West Africa to examine the role of these interactions on tropical ecosystem stability. We find that increased aridity between 28–15 kyr B.P. led to the widespread expansion of tropical grasslands, but that frequent fires and low CO_2_ played a crucial role in stabilizing these ecosystems, even as humidity changed. This resulted in an unstable ecosystem state, which transitioned abruptly from grassland to woodlands as gradual changes in CO_2_ and fire shifted the balance in favor of woody plants. Since then, high atmospheric CO_2_ has stabilized tropical forests by promoting woody plant growth, despite increased aridity. Our results indicate that the interactions between climate, CO_2_ and fire can make tropical ecosystems more resilient to change, but that these systems are dynamically unstable and potentially susceptible to abrupt shifts between woodland and grassland dominated states in the future.

Savanna-forest complexes occupy nearly 20% of the Earth’s land surface^1^, are responsible for 30% of the Earth’s terrestrial primary production and play an important role in global carbon budgets^2^. Anthropogenic climate change is anticipated to have a significant influence on the ecology and distribution of tropical ecosystems, though the magnitude, rate and direction of these changes are uncertain^3^. These uncertainties reflect the competing influences of moisture availability, CO_2_ and fire on tropical vegetation^4^,^5^,^6^. For example, although the dominant factor controlling the potential amount of woody cover in savanna ecosystems is precipitation, actual woody cover is controlled by landscape disturbance, much of which is attributable to fire^7^,^8^. The establishment of woody plants in fire-prone tropical grasslands depends on both the frequency of fires and growth rates by affecting whether plants can achieve sufficient stem diameters and heights to survive subsequent fires^9^,^10^. In ecosystems with frequent fires or slow growth rates, woody plants may be unable to escape this “fire trap” despite increases in moisture availability. Changes in CO_2_ may alter these relationships by changing the carbon available for woody plant growth^9^,^10^, and increased CO_2_ has been shown to increase growth rates, leading to increased survivability in the presence of fire^9^,^11^,^12^.

To better understand the stability of tropical forest-savanna systems, we use the sediment record from Lake Bosumtwi, Ghana, which is located in the lowland tropical forest, several hundred kilometers south of the modern-day savanna-forest boundary (Fig. 1). We reconstruct changes in vegetation and hydrology using the carbon and hydrogen isotope composition of sedimentary leaf wax *n-*alkanes (δ^13^C_wax_, δD_wax_). Changes in δ^13^C are interpreted as reflecting changes in the relative proportions of plants using the C_3_ and C_4_ photosynthetic pathways^13^. C_3_ vegetation includes most woody plants and winter season grasses with average δ^13^C values that are as low as −36‰, whereas most summer or dry season C_4_ grasses have δ^13^C values of around −20‰^14^. We simultaneously reconstruct changes in precipitation using hydrogen isotope analysis of the same compounds (δD_wax_)^15^, which allows us to assess the relative phasing of vegetation and precipitation changes independent of any potential complications associated with organic matter transport pathways or residence time.

Changes in fire frequency are estimated from layer counts of distinct charcoal laminae within the annual sediment laminations (Fig. 2). Because the charcoal occurs as layers and is composed mostly of relatively intact fragments, we interpret the charcoal layers as reflecting the seasonal transport of charcoal to the lake from dry season fires with little or no time lag. We then use the frequency of charcoal layers as a proxy for annual fire frequency. Reconstructions of fire frequency are supported by fire reconstructions from concentrations of levoglucosan, a byproduct of cellulose combustion, and sediment particulate charcoal counts in pollen slides. Previous studies have shown that particulate charcoal^16^ and levoglucosan^17^ can provide reliable indicators of paleofire in some sedimentary archives and Lake Bosumtwi is an ideal location for preservation of the fire marker levoglucosan because of its permanent bottom water anoxia and well-preserved organic matter^18^. While all three approaches provide proxies of changes in fire regime, processes such as transport and deposition, fire intensity/temperature and fuel source will affect them differently, leading to differences in fire reconstructions^19^,^20^,^21^,^22^,^23^ (Fig. 3). However, the most significant changes are consistent between the fire proxy indicators, indicating that they do preserve consistent large scale variations in the fire regime (SOM).

## Results

### Evidence for a nonlinear tropical vegetation response to changes in hydroclimate

**Figure 3:**
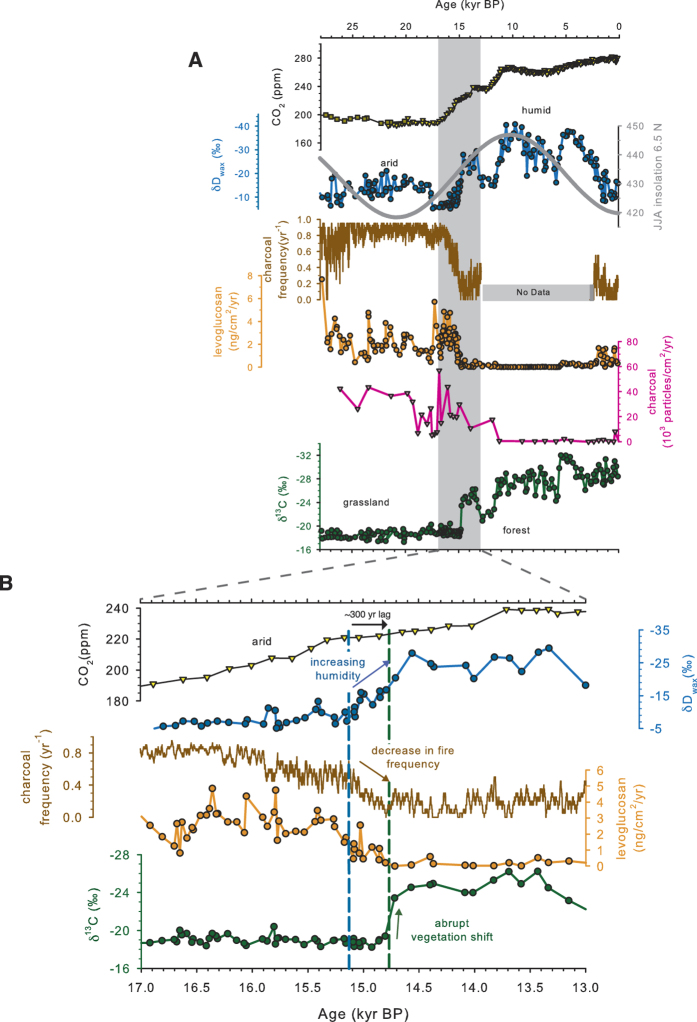
(**A**) Temporal variations in the climate-fire-CO_2_-vegetation system of tropical West Africa over the last 28,000 years BP reconstructed from Lake Bosumtwi. Atmospheric CO_2_ from the EPICA (triangles)^42^ and Taylor Dome ice cores^43^ (squares). Computed changes in Northern Hemisphere summer (JJA) insolation at 6.5°N^44^ (grey line). Reconstructed precipitation changes from the δD values of C_31_ *n*-alkanes (blue circles) from Lake Bosumtwi^15^. Reconstructed changes in fire from a 20-year moving average of the frequency of charcoal layers in thin sections (brown), concentrations of the cellulose combustion byproduct levoglucosan (ng/cm^2^/yr) orange circles) and particulate charcoal influx (particles/cm^2^/yr; pink diamonds). Relative proportion of C_3_ and C_4_ plants from the δ^13^C values of C_31_ *n-*alkanes. (**B**) Expanded view of the transition from dry, frequently burned, grassland dominated ecosystems to more humid, low fire and CO_2_ dominated forested ecosystems during the last deglaciation. The blue dashed line indicates the onset of humid conditions, the green dashed line indicates the midpoint of the abrupt deglacial vegetation shift.

The Lake Bosumtwi record shows that the climate, fire and ecological regimes of tropical West Africa changed significantly over the past 28,000 years. During the early part of the record (Fig. 3A; 15,000–28,000 yr BP), δD_wax_ values were positive, indicating more arid conditions, fire was more frequent and δ^13^C_wax_ values were higher (−17.3 to −20.6‰) indicating that the landscape was dominated by drought-tolerant C_4_ summer grasses with only minor contributions from C_3_ woody plants that burned almost annually. In comparison with the rest of the record, δ^13^C_wax_ values show little variability, despite significant variations in δD_wax_ and fire at this time. After ca. 15,100 yr BP (Fig. 3B, blue dashed line), more depleted δD_wax_ values indicate a gradual return to more humid conditions. These are accompanied by a decline in the frequency of fires, which disappear almost entirely for much of the most humid portion of the Holocene (i.e., 10,000–3,200 yr BP). However, in contrast to the gradual nature of these changes in precipitation and fire, the δ^13^C_wax_ record suggests that the vegetation response was abrupt, with a ~25% change in the proportion of C_3_ woody plants in <150 years. The rapid expansion of woody plants also occurs ca. 300 years after the onset of wetter conditions (at 14,800 yr BP), at the time when fire frequency had declined from nearly annual to one fire every five years (Fig. 3B, green dashed line). Over much of the remainder of the record, the landscape around Lake Bosumtwi was dominated by a variable mix of C_3_ and C_4_ plants suggestive of an open woodland or forest environment similar to today, though the data also suggest a trend towards an increasing proportion of C_3_ plants over the past 14,500 years. Coherent variations in δ^13^C_wax_ and δD_wax_, during this period indicate that the dominant control over changes in the relative proportions of C_3_/C_4_ plants was precipitation, as expected in the absence of fire disturbance. After ca. 3200 yr BP, δD_wax_ values suggest that conditions became significantly more arid, and were accompanied by an increase in fire. Nevertheless, fire frequency during the late Holocene was much lower than during the late Pleistocene (27 to 15 ka), and the magnitude of the fire response recorded in the levoglucosan and particulate charcoal records differ, suggesting a distinctly different fire regime. Unlike the earlier part of the record, severe late Holocene aridity (∆δD_wax_ = ~17‰) was associated with only minor changes in δ^13^C_wax_, (∆δ^13^C_wax_ = ~2–3‰) indicating that woody plant-dominated ecosystems persisted despite these environmental changes.

The changing relationships between δ^13^C_wax_ and δD_wax_ suggest that reconstructed vegetation changes over the past 28,000 years cannot be explained solely by precipitation but instead reflect the competing influences of climate, fire and atmospheric CO_2_, as suggested by modeling and modern ecosystem studies^24^,^25^. The invariant nature of the early portion of the δ^13^C_wax_ record, when compared with changes in δD_wax,_ shows that these grasslands were insensitive to temporarily increased precipitation. The fire proxies also show that at this time, significant landscape-scale fires occurred at annual to interannual timescales. Our evidence for extensive grasslands across tropical Africa at this time with a high frequency of burning is supported by both vegetation synthesis and modeling studies^26^,^27^ and lower resolution fire reconstructions from marine sediment cores, which indicate that glacial conditions were accompanied by substantial export of fire-derived elemental carbon^28^. We hypothesize that these fires were sufficiently frequent and widespread to prevent woody plants from becoming established, as can occur in modern savannah ecosystems^7^,^8^. Prior to ~15,000 yr BP, this effect would have been exacerbated by low CO_2_ levels, which would have slowed the rate of woody plant germination, growth and recovery, making it difficult for C_3_ plants to recover from fire disturbance even as humidity increased^4^.

### A critical role for fire and CO2 in controlling tropical vegetation stability

**Figure 4:**
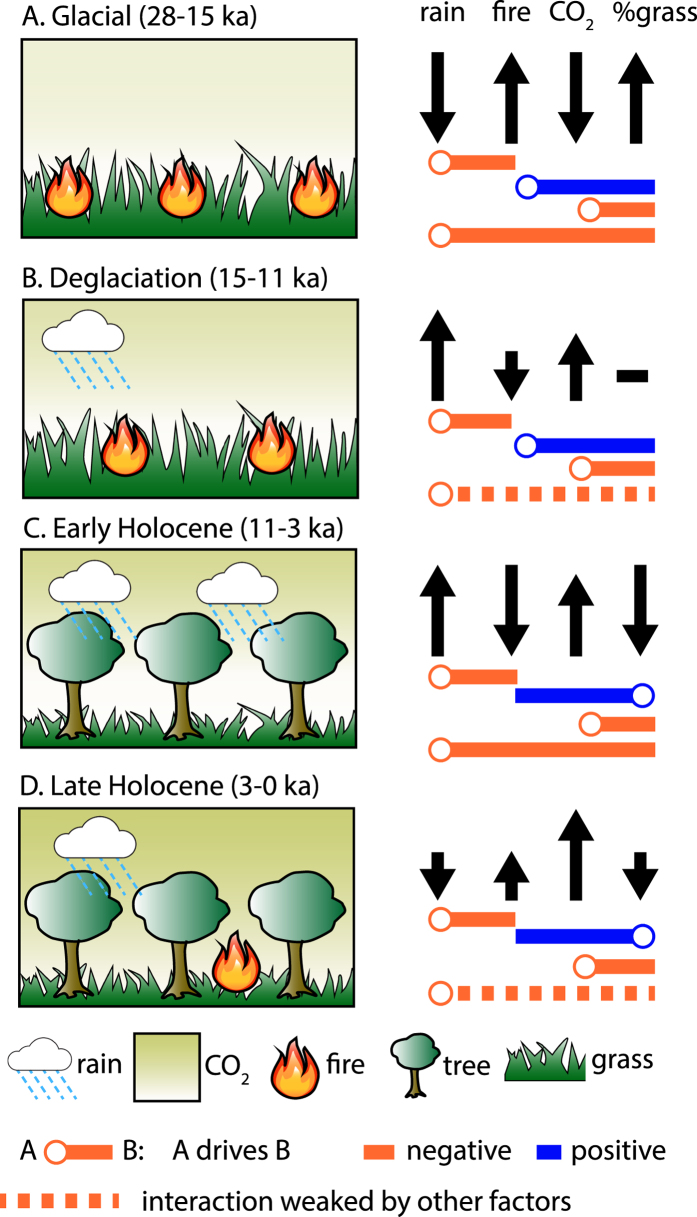
Model of the impacts of climate, fire and atmospheric CO_2_ on the vegetation of tropical Africa. Left: cartoon of changes in the climate-ecosystem balance as a function of time. Right: schematic of changes in each parameter indicating the size and direction of change between timesteps (arrows) and the relationships between the parameters (red: negative feedback, blue: positive feedback, circle: hypothesized driver of the relationship, dashed: the relationship between these two parameters is weakened by other feedback relationships.

We propose that the high fire frequency early in the record is also the proximate cause of the abrupt and delayed expansion of C_3_ plants at 14,800 yr BP (Fig. 4). The rapid shift in vegetation occurred when precipitation was similar to earlier (>18,000 yr BP) and later (<3000 yr BP) periods when no systematic vegetation change happened, demonstrating that vegetation was not responding to changes in precipitation alone (Fig. 3). Instead, the abrupt vegetation change reflects a threshold response to the interactions between gradually declining fire frequency, increasing CO_2_ and increasing precipitation. The abruptness of this transition likely reflects a combination of the equilibration of C_3_ plant composition with available moisture and a feedback between woody plant expansion and a further reduction in the spread of subsequent grassland fires. Support for a threshold mechanism in controlling the vegetation response is also consistent with the ~300 year lag between the initial changes in δD_wax_ and δ^13^C_wax_, and the transition from a system where vegetation is unresponsive to changes in precipitation (>14,800 yr BP) to one where the dominant control on vegetation is precipitation (14,800–3,000 yr BP) (Fig. 3). Although other abrupt changes in vegetation occur in the record at ca. 11,500 yr BP and 5700 yr BP, these lack the characteristics indicative of a threshold vegetation response (Supplementary Information Fig. S4). For example, neither show clear evidence for a lag in the timing of the vegetation response to climate, nor is there evidence of changing sensitivity of the vegetation to environmental conditions. Both of these are evident in the shift at 14,800 yr BP.

Following the initial expansion of C_3_ plants and for much of the remaining record, the frequency and intensity of fire remained low, CO_2_ was high and increasing, and vegetation responded on centennial to millennial timescales to changes in precipitation, with greater proportions of C_3_ plants accompanying wetter conditions (Fig. 3). However, this interval is also characterized by an overall trend towards greater C_3_ plant abundance and a decrease in the sensitivity of vegetation to changes in precipitation. We hypothesize that this reflects the influence of increasing CO_2_, which favors the growth of C_3_ plants relative to C_4_ grasses^29^. These effects would also have been enhanced by biogeophysical feedbacks; well-developed forests can act to suppress the growth and colonization of grasses by reducing ground light availability, maintaining higher sub-canopy humidity and keeping temperatures lower, all of which promote the growth of C_3_ plants and are capable of maintaining forest stability^30^, even as environmental conditions change.

## Discussion

The Lake Bosumtwi record highlights the competing roles of climate, fire and CO_2_ on the long-term stability and evolution of grasslands and forests of tropical West Africa. In agreement with many studies of modern tropical grassland ecosystems, frequent fires played a crucial role in excluding woody plants during glacial to late glacial times, even as precipitation and CO_2_ increased^4^,^5^,^6^,^9^,^10^,^31^ (Fig. 4). However, this also resulted in a state of disequilibrium between climate and vegetation, leading to an abrupt vegetation response to changes in climate. In contrast, dramatically increased aridity and fire in the late Holocene did not cause a substantial increase in drought-tolerant C_4_ grasses, suggesting that tropical forests became more resilient to changes in climate and disturbance. We propose that this is a consequence of high CO_2_ and biogeophysical feedbacks, which resulted in conditions more favorable for C_3_ plant growth and recovery, stabilizing existing forest cover despite the increased aridity (Fig. 4). We hypothesize that, as with the late glacial grasslands, this has resulted in a dynamically unstable climate-vegetation state that is susceptible to rapid transitions between C_3_ and C_4_ plant-dominated landscapes^32^. Although elevated CO_2_ should continue to act as a stabilizing element, at higher CO_2_ the advantages for C_3_ plants decrease^33^, limiting its ability to stabilize these systems and increasing the potential for an abrupt collapse of tropical forest ecosystems in response to increased aridity. The sensitivity of these ecosystems to slight perturbations in climate today is evident in the Dahomey Gap, just to the east of Lake Bosumtwi, where precipitation is only 200–400 mm yr^−1^ lower than Bosumtwi but the natural landscape is dominated by grasslands with only isolated patches of forest^34^. Here, vegetation records indicate that small increases in rainfall resulted in wholesale return of forest cover briefly in the late Holocene^35^. In the future, much of West Africa could become more arid, provided that projected increases in precipitation are insufficient to overcome a net drying of soils driven by increased temperature^36^. Such gradual changes in hydroclimate and fire could potentially overwhelm the natural buffering capacity of high CO_2_ and allow current climate-vegetation stability to give way to rapid forest-savanna transformation in a region that is already a mosaic of these two land-cover types.

## Methods

### Study site

Past variations in the climate, vegetation and fire history of West Africa were reconstructed using the sediment geochemical record from Lake Bosumtwi, Ghana. Lake Bosumtwi is a small (~8 km diameter), internally draining lake occupying a meteorite impact crater in southern Ghana (Supplementary Fig. S1). The lake is deep (~75 m depth asl) and sheltering by the crater walls inhibit mixing, leading to stratification and permanently anoxic bottom waters. This limits bioturbation and allows for the preservation of mm-scale laminations that have been demonstrated to be annual^18^. The majority of the catchment is forested except for the flat lying terraces and drainages, which have been converted to agriculture^37^. The regional climate is controlled primarily by the West African monsoon, which brings moisture to southern Ghana in May-September when the Intertropical Convergence Zone (ITCZ) migrates to the north (Supplementary Fig. S1). In winter, as the ITCZ shifts southward, the climate of the region is dominated by dry northwesterly winds, which inhibit rainfall.

### Age model development

A detailed description of the age model used in this study is published elsewhere^38^. It is based on a combination of radiocarbon dating, Bayesian age depth modeling and varve counting. The chronology consists of 107 radiocarbon dates on bulk organic matter and macrofossils covering the upper 21.4 meters of sediment. Dates from different cores were correlated using marker laminae matches and Bayesian age depth modeling was performed using the R software package BACON^39^ and using the IntCal09 radiocarbon calibration curve^40^. Over the deglacial section of the core, we revised the age model by wiggle matching the uncalibrated radiocarbon ages to the IntCal09 calibration curve using the varves as a constraint on the time elapsed between radiocarbon age. An optimal fit was determined using Χ^2^ minimization^38^.

### Lipid and compound-specific stable isotope analysis of n-alkanes

Sediment samples (1–5 g) were freeze dried, homogenized and solvent-extracted by either accelerated solvent extraction (Dionex ASE 200) or a microwave extraction (MARS) using dichloromethane:methanol (9:1; v/v). *n-*alkanes and levoglucosan were isolated from the total lipid extract by silica gel column chromatography. *n-*alkanes were additionally isolated by silver nitrate silica gel chromatography and molecular sieve or urea adduction. Stable carbon and hydrogen isotope analysis of the long chain n-alkanes was performed by gas chromatography isotope ratio mass spectrometry (GC-IR-MS). Description of the methods for δD_wax_ analysis and corrections for changes in vegetation and ice volume are described in the online Supplementary Materials. δ^13^C_wax_ values were measured against internal calibrated propane reference gas standards and are reported in % VPDB (Vienna Pee Dee Belemnite). The precision of the analysis, based on repeated analysis of the standard mix was better than 0.3%. Each sample was measured in triplicate and the mean precision for the long chain *n-*alkanes (C_27_, C_29_, C_31_, C_33_) based on this replicate analysis was better than 0.4%. Levoglucosan analysis was performed by gas chromatography mass spectrometry. Identification of the trimethylsilyl ether derivative of levoglucosan was identified in the mass scan by its characteristic fragments (m/z 363, 333, 217, 204) and its retention time determined with an authentic standard. Quantification was performed using an internal standard (androstanol). Levoglucosan concentrations (ppm) were normalized to time (ppm/yr) using varve counts over the laminated intervals of the core and using the Bacon age-depth model over the unlaminated sections.

### Reconstruction of fire frequency from sedimentary charcoal

Paleofire was reconstructed from a combination of microscopic particulate charcoal counts and frequency analysis of charcoal layers visually identified in sediment thin sections under transmitted light (Supplementary Fig. S2). Particulate charcoal fragments above 10 μm were counted on slides prepared for palynological analysis. A minimum of 2000 charcoal particles were counted per sample and abundance relative to an exotic marker, *Lycopodium*, was calculated. Charcoal counts were normalized to time (particles/cm^2^/yr) using the same approach as for levoglucosan. Fire frequency was estimated using visual identification of annual charcoal layers in sediment thin sections under transmitted light. Previous work on the laminations from Lake Bosumtwi has demonstrated that they are annual in nature and visible charcoal occurs as a distinct layer within many of the annual laminations, suggesting that the charcoal is deposited after seasonal fire events, rather than being remobilized material from fires occurring in previous years. Charcoal layer frequency analysis was performed on a single continuous set of thin sections spanning the record. Reproducibility was assessed via replication over a several short, overlapping intervals.

## Additional Information

**How to cite this article**: Shanahan, T. M. *et al*. CO_2_ and fire influence tropical ecosystem stability in response to climate change. *Sci. Rep.* **6**, 29587; doi: 10.1038/srep29587 (2016).

## Supplementary Material

Supplementary Information

## Figures and Tables

**Figure 1:**
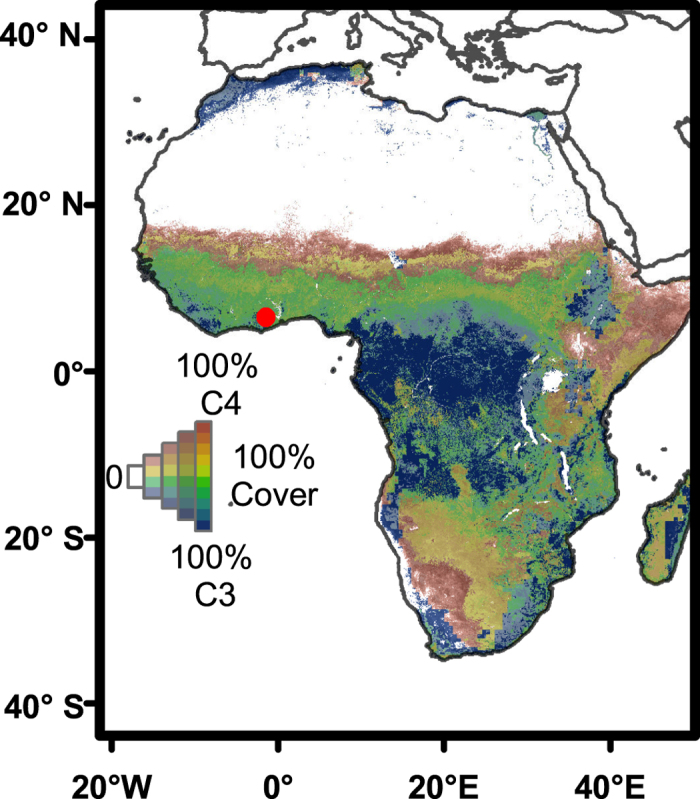
Present-day distribution of vegetation across Africa. Modeled variations in vegetation cover computed as proportions of C_3_ and C_4_ plants replotted from^41^. White areas indicate deserts. Circle indicates the location of Lake Bosumtwi, Ghana (6°30′N, 1°25′W). Map produced using ESRI ArcGIS software [v.10.3.1], (http://www.esri.com/software/arcgis).

**Figure 2:**
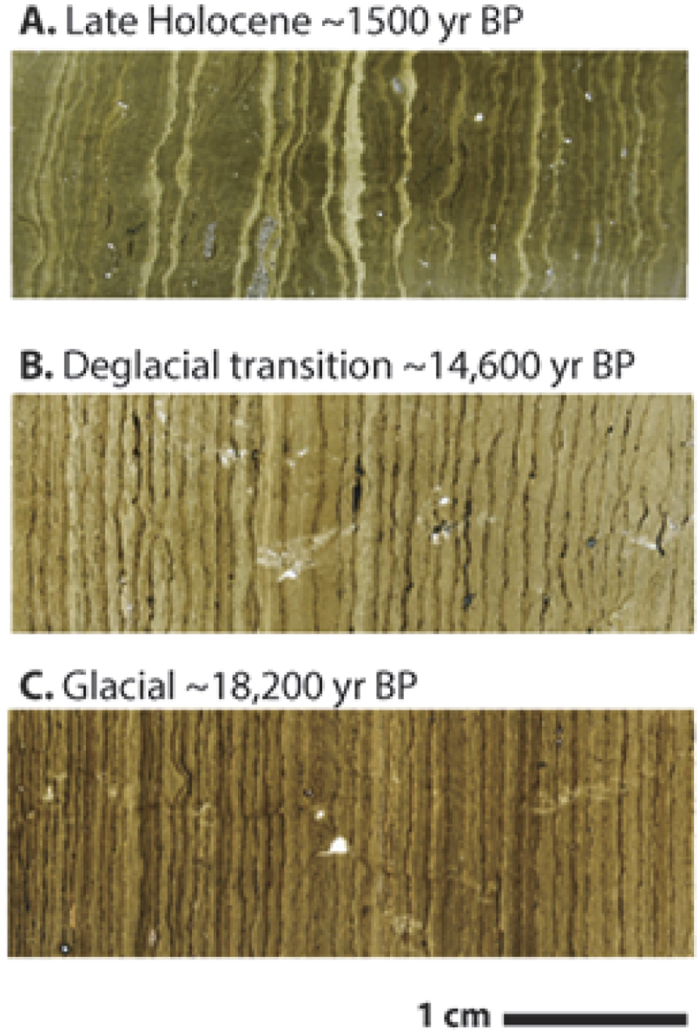
Evidence for changes in fire frequency. Photomicrographs showing changes in the frequency of charcoal laminations in transmitted light sediment thin section from (**A)** the late Holocene (~1500 yr BP) when discrete bands of charcoal are absent, (**B)** the deglacial transition (~14,600 yr BP) when charcoal frequency has started to decline, (**C)** the glacial (~18,200 yr BP) when charcoal bands occur almost annually. Varves are evident in the millimeter to sub-millimeter scale couplets of light and dark laminae. The charcoal appears as distinct black layers in the boundaries of some of the annual laminae.
