# Supplementary material for: CO2 and fire influence tropical ecosystem stability in response to climate change
Source: Sci Rep. 2016 Jul 18;6:29587. doi: 10.1038/srep29587 (PMC4947926; doi:10.1038/srep29587)
Supplement: Supplementary Information [file srep29587-s1.pdf]

Supplementary Information

CO_2 and fire influence tropical ecosystem stability in response to climate change

Timothy M. Shanahan, Konrad A. Hughen, Nicholas P. McKay, Jonathan T. Overpeck, Christopher A. Scholz, William D. Gosling, Charlotte S. Miller, John A. Peck, John W. King, Clifford W. Heil

1. Sediment core collection

The cores studied here were obtained during visits to the lake in 1999 and 2004 and were collected from the center of the lake, away from any river inputs. Freeze cores were used to capture the sediment-water interface and were correlated to the longer piston cores using marker laminations. Drill cores were obtained under the Intercontinental Drilling Program (ICDP) drilling project in 2004, and provide a continuous record of sedimentation going back to the initial formation of the crater at 1.07 Ma [1]. This study focuses on the uppermost 10.6 meters of this sediment sequence preserved in the piston cores, which covers the last 28 kyr.

2. Supplementary Methods

2.1 Biomarker analysis

2.1.1 *n*-alkane extraction and separation

Total lipid extracts (TLEs) were obtained by solvent extraction of 1-5 grams of freeze dried sediments using either a Dionex Accelerated Solvent Extraction System or a CEM MARS X Microwave Extraction System with dichloromethane:methanol (9:1; v/v). Separation of the total lipid extract into acid and neutral fractions was performed by aminopropyl flash chromatography using 9:1 DCM:MeOH and 2% formic acid in DCM. The neutral fraction concentrated under N_2 and separated into polar and apolar fractions over silica gel with hexane and MeOH. Branched compounds and alkenes were removed from the hydrocarbon fraction by urea adduction or molecular sieve and when needed, Ag^+ chromatography prior to stable isotope analysis.

2.21.2 Compound specific $\delta^{13}C$ analysis of n-alkanes

Carbon isotope analysis of individual *n*-alkanes was determined using an Agilent 6890 gas chromatograph operated in splitless mode and equipped with a DB-5 µs column (30m x 0.25µm x 0.25mm), coupled to a Delta V isotope ratio mass spectrometer via a Finnigan GC/C III combustion interface operated at 960°C. Instrument operation was assessed daily using a standard containing six *n*-alkanes of known isotopic composition measured repeatedly. The precision of the analysis, based on repeated analysis of the standard mix was better than 0.3 ‰. Each sample was measured in triplicate and the mean precision for the long chain *n*-alkanes (C_{27}, C_{29}, C_{31}, C_{33}) based on this replicate analysis was better than 0.4‰. Hydrogen isotope analysis was performed as described previously[2]. Standard deviations of δD values for the C_{31} n-alkanes based on replicate analysis of the same samples were typically <±3 ‰. Reported hydrogen isotope values have been corrected for changes in source associate with ice volume and vegetation, as described previously[2].

2.1.3 Identification and quantification of levoglucosan

Source-specific organic geochemical markers of fire offer an alternative means of reconstructing changes in fire frequency or intensity that have the advantage in that their analysis can be coupled with more traditional biomarker analysis. One such marker, used here, is levoglucosan, a monosaccharide anhydride, which is derived only from the pyrolysis of cellulose, making it an excellent tracer of natural fires [3]. Previous work on a sediment record from the Amazon showed an excellent correlation between levoglucosan concentrations and charcoal, suggesting that levoglucosan can provide a similar record of past fire, albeit at potentially much higher resolution [4].

Mass spectral analysis of levoglucosan concentrations on selected samples were performed on either fractions isolated by silica gel chromatography, or on aliquots of the total lipid extracts (TLEs; n = 192 samples). Prior to analysis, all samples were silylated using BSTFA +1% TMCS and pyridine (60°C, 30 minutes). Analyses were performed using a

ThermoQuest TRACE gas chromatograph with a PAL autosampler and a PTV injector coupled to a Thermo Electron Inc. Tempus Time of Flight- mass spectrometer. The PTV injector was run in solvent split mode with a constant septum purge and a 0.4 ml/min He gas flow. The GC was equipped with a 10 meter (0.10 mm x 10mm) Restek Rtx-1 column and the mass spectrometer was operated at an acquisition rate of 20 spectra/second. Initial temperature was held at 40°C for 3 minutes after which the temperature was ramped to 120°C at 75°C/min and 320°C at 37C/min and maintained at 320°C for 4 minutes. The trimethylsilyl ether derivative of levoglucosan was identified in the mass scan by its characteristic fragments (m/z 363, 333, 217, 204) and its retention time as determined with an authentic standard (obtained from Sigma, St. Louis, MO, USA). Concentrations were determined by a correction factor which was determined offline using the internal standard (androstanol) added to each TLE immediately after solvent extraction. Levoglucosan concentrations were normalized by the time of deposition using either varve counts or, in the unlaminated sections, age depth model estimates of sedimentation rates and are presented as $ng/cm^2/yr$.

2.2 Charcoal analyses

2.2.1 Particulate charcoal analysis

Estimates of charcoal concentration were made by manual counts of charcoal particles >10 um in diameter on pollen slides under 400x magnification with transmitted light (n=37 samples). Charcoal fragments were identified by their angular structure, opacity and the presence of visible stomata[5]. Charcoal concentration values were calculated by adding a spike of 1 *Lycopodium* spore tablet to samples of known volume[6]. To ensure sufficient precision was reached a minimum of 2000 charcoal fragments were counted per sample [7] by comparison with *Lycopodium* counts. Total charcoal concentrations were normalized by the time of deposition using either varve counts or, in the unlaminated sections, age depth model estimates of sedimentation rates. Charcoal data is presented as $particles/cm^2/yr$.

2.2.2 Charcoal laminae frequency analysis

Charcoal frequency analysis was performed on visual identifications of charcoal layers in sediment thin sections examined under transmitted light. Identifications were based on distinct, dark colored sublaminae. In thin sections, the charcoal appears as a single, distinct layer within each annual lamination – consistent with seasonal delivery of charcoal to the basin in conjunction with dry season fires. A limited number of checks (n = 132) were performed on sediment samples throughout the record to confirm that these darker layers were composed of charcoal (Fig. S2), though it was not possible to provide regular checks on individual laminae. We attempted to quantify the amount of charcoal within the laminae using the thickness/area of the charcoal layers in thin section photographs, but the results were not reproducible across thin sections from different cores. In contrast, the identification of charcoal layers in thin section was reproducible across thin sections of overlapping cores. For the older portion of the record (28,000-12,800 yr BP) the reproducibility of the charcoal layer identifications was >85%. For the uppermost ~2 millennia, the reproducibility was also good (> 70%). However, for the interval between ~2000-2700 yr BP it was too difficult to accurately identify charcoal (reproducibility <45%) so no data are reported. In addition, no charcoal layer frequency data is reported for the interval between 2700-12,800 yr BP because this section of the sediment record is massive and unlaminated. For all reported data, charcoal frequency is reported based on a 20 year running average in order to smooth out locally significant intervals of high and low charcoal frequency. The choice of averaging interval does not significantly change the results.

3. Comparison of charcoal and levoglucosan data

To test the effectiveness of levoglucosan as a proxy for fire, we directly compared levoglucosan concentrations to microscopic charcoal counts. Since charcoal counts were performed separately on slides prepared for pollen analysis, lipid and charcoal data were not available from exactly the same depths. To compare the lipid and charcoal data directly, we linearly interpolated

the levoglucosan data to the same depths as the charcoal samples. The charcoal and levoglucosan data were significantly correlated ($p=0.0009$, $n= 36$), though the charcoal data was found to be only moderately good predictor of the levoglucosan concentrations ($R=0.53$) (Fig. S3). Considering the sample-to-sample variability and that the levoglucosan data were interpolated to the depths of the charcoal data, the relatively low correlation coefficient is not surprising. However, the overall similarities in the deglacial scale changes in charcoal and levoglucosan is evident (main text Fig. 3) and occurs in conjunction with the reduction in fire frequency from charcoal laminae analysis.

Several factors may explain some of the differences between the variations in levoglucosan and charcoal. First, levoglucosan is likely to represent a more regional signal of combustion, given that it is known to be transported over very long distances [8,9]. Transport and dispersal also plays an important role in charcoal records but the transport distances are likely much shorter for the charcoal than the levoglucosan [10-14]. Combustion temperature may also play a role in the differences between the levoglucosan and the charcoal records, with significant production of levoglucosan occurring at even very low temperatures (<300°C) [15]. However, both charcoal and levoglucosan production may also be influenced by fuel type [15-17]. Intervals where the response of the charcoal and the levoglucosan proxies differ could be due to any combination of these factors. As a result, we focus here on the overall similarities in these proxies as an indication of overall changes in the fire regime.

4.Estimating the proportion of C_3 and C_4 plants from leaf wax $\delta^{13}C$ values.
In order to reconstruct vegetation changes with the same temporal resolution and to avoid any potential complications associated with differences in vegetation proxy (e.g., pollen vs. plant wax) residence times, we utilized changes in the $\delta^{13}C$ of the plant waxes as a proxy for changes in the proportion of C_3 and C_4 vegetation. Most woody vegetation (e.g., trees and shrubs) utilize the Calvin-Benson (C_3) carbon fixation pathway, whereas summer grasses use the Hatch-Slack (C_4) cycle [18-20]. Since these photosynthetic pathways result in different

carbon isotope fractionation, $\delta^{13}C$ values can provide estimates of the relative proportions of grassy and woody vegetation present at a site. This approach has been used in a number of studies to estimate past changes in vegetation cover in tropical Africa (e.g.,[21-23], assuming a linear mixing model of endmember $\delta^{13}C$ compositions for C_3 and C_4 plants.

A number of studies have recently raised concerns about the use of $\delta^{13}C$ to estimate vegetation composition [24,25]. Factors that may affect the appropriateness of this approach include species-specific differences in leaf wax production [26], the fact that C_3 and C_4 plants can display a range of endmember $\delta^{13}C$ values [24] and that factors other than vegetation type (e.g., aridity, CO_2, canopy cover) may affect the degree of carbon isotope fractionation, particularly in C_3 plants [19,24,27-30]. In addition, a recent study of the $\delta^{13}C_{wax}$ values of modern plants and lake surface sediments from Cameroon suggested that linear mixing models may not be appropriate for some environments, biasing vegetation reconstructions [25].

In the present study, we use a linear mixing model to estimate changes in vegetation from $\delta^{13}C_{wax}$. We revised our calculations from our previous work [2] using the maximum $\delta^{13}C$ value in our dataset as an estimate of the $\delta^{13}C$ value of the C_4 endmember (-17.28‰; close to the value of -17.7‰ previously estimated from modern African vegetation data[25]). For the $\delta^{13}C$ endmember value of C_3 plants, we used the estimate of -33.8‰ from modern African vegetation surveys[25], which is slightly lower than the minimum value obtained in our dataset (-31.9‰). Based on these values we estimate that the vegetation cover ranged from 100% C_4 grasses during the earlier part of the record to as much as 89% C_3 vegetation during the wettest parts of the Holocene. Using the published nonlinear mixing models[25] produces results with a similar total range (e.g., 0-80% C_3 vegetation) but average vegetation cover during the Holocene has a much lower proportion of C_3 plants (34 and 39% using the sine-squared and hyperbolic models versus 68% using the linear model) and a higher proportion of C_4 plants prior to 15 kyr (100, 97% versus 91%). Model estimates are broadly consistent with published pollen data[31] for the earlier part of the record with *Graminaea* and

Cyperaceae pollen reaching values as high as 93% between 18-21 kyr BP, though the pollen data shows overall much greater variability than the $\delta^{13}C_{wax}$ during this interval. In the Holocene, abundances of arboreal plant taxa estimated from pollen data are around 83%[31]. Although these data suggest that the linear model underestimates the proportion of C_3 plants, the linear model is much more consistent with the pollen data than is either of the nonlinear models proposed for West Africa[25]. For this reason, we chose to employ the linear model for estimating vegetation changes in this study.

5. Adjustments of n-alkane hydrogen isotope values for changes in global ice volume and vegetation.

Leaf wax hydrogen isotope data are interpreted here as reflecting changes in aridity. This is related to two factors: (a) the observation that in much of the tropics, including tropical West Africa, increasing rainfall amount is correlated with increasingly negative hydrogen and oxygen isotope values for precipitation ([32]; and (b) that some plants display more positive hydrogen isotope values in their waxes under more arid conditions, as a result of leaf wax synthesis using leaf water that is more positive isotopically because of increased rates of evapotranspiration and kinetic fractionation of leaf waters [33]. Because these effects act in the same direction (wetter = more negative; drier = more positive), they reinforce one another, enhancing the aridity signal.

However, these are not the only factors affecting the hydrogen isotope composition of leaf waxes. The precipitation source can be affected by a variety of factors such as moisture source pathway and changes in the stable isotope composition of the oceans from which the precipitation is derived. Because of the proximity of our site to the Gulf of Guinea, it is unlikely that changes in moisture pathways had a significant influence on the isotopic composition of rainfall over the interval studied here. However, given the changes in global ice volume and their effect on the stable isotope composition of the oceans since the last glacial maximum, we have corrected our leaf wax data for this effect. Our corrections follow the approach used by previous workers [34] and presented in our

previous work on Lake Bosumtwi [2]. Briefly, we assume a 1‰ change in $\delta^{18}O$ at the Last Glacial Maximum [35,36] and convert this to the δD value of the LGM ocean by assuming a deuterium excess of zero [34,37]. We then generated estimates of the δD of the ocean for each leaf wax isotope measurement using the LR04 benthic oxygen isotope stack [38] and used these values to remove the effect of ice volume changes from the leaf wax data. Overall, the effect of this correction was to reduce the magnitude of the glacial-Holocene hydrogen isotope shift in the leaf wax reconstruction (Fig. S4a).

Hydrogen isotope values of leaf waxes are also influenced by biosynthetic fractionation during leaf wax synthesis, which produces a substantial negative offset in the leaf wax hydrogen isotope values from their source water [33]. However, the magnitude of this offset differs between plant types [39-46] complicating the interpretation of leaf wax hydrogen isotopic data, particularly in records containing significant changes in vegetation, like Lake Bosumtwi. Because high-resolution pollen data is not available, we used $\delta^{13}C_{wax}$ as a proxy for the proportion of C_3 and C_4 plants (assuming a linear mixing of two endmembers, sec.4) [47]. We used estimates of the endmember apparent hydrogen isotope fractionation factors for C_3 and C_4 plants (-125±5‰[48] and -145±15‰[49], respectively) to compute an effective apparent fractionation factor for each sample and corrected the ice volume corrected δD_{wax} values accordingly. This correction increases the magnitude of the deglacial hydrogen isotope shift (Fig. S4b). And the magnitude of this shift is strongly dependent upon both the endmember values used for the apparent hydrogen isotope fractionation in C_3 and C_4 plants and the estimates of C_3 and C_4 plant percentages from $\delta^{13}C_{wax}$ values. The revised estimates of plant composition used here result in slightly more positive reconstructed hydrogen isotope values for that interval. However, the corrections to the plant wax hydrogen isotope data do not substantially change the abruptness of the aridity transitions (Fig. S5) and the major climate transitions in the revised δD reconstructions remain significantly more gradual than the reconstructed vegetation shifts in the $\delta^{13}C$ data (Fig. S6;section 6).

6. Comparisons of the relative abruptness and timing of the major vegetation-climate shifts.

In this paper, we argue that the abrupt vegetation shift during the deglaciation is unique, and reflects a delayed threshold response of the vegetation to more gradual increases in climate (from δD_{wax}) and decreases in fire frequency. This is based upon the fact that deglacial changes in $\delta^{13}C_{wax}$ (i) occur abruptly (<150 years) than the corresponding changes in δD_{wax} and fire frequency; and (ii) that they lag the changes in δD_{wax} and fire by ca. 300 years. Based upon their association with gradually declining fire frequency and the dynamics of modern fire-prone savanna ecosystems, we argue that the reduction in fire, in combination with gradually increasing atmospheric CO_2, created a threshold, nonlinear vegetation response to gradually increasing rainfall.

However, there are also abrupt changes in vegetation at both the end of the Younger Dryas and in the mid Holocene, when jumps in $\delta^{13}C_{wax}$ values suggest sudden expansions of C_3 vegetation (Text Fig. 3a). We compare these transitions in Fig. S6a-c. From this comparison it is evident that the vegetation shift at the end of the Younger Dryas, though rapid, is synchronous with and follows a similar, gradual trend as the changes in hydrology inferred from δD_{wax}, (Fig S6c). This suggests a linear vegetation response to changes in climate at that time, in contrast to the response during the deglaciation.

In contrast, the mid-Holocene vegetation change is as large as the one during the deglaciation, and could possibly be as abrupt, given the differences in sampling density (Fig. S4b). We argue that the deglacial vegetation change is fundamentally different from the mid-Holocene change, and does not reflect a threshold vegetation response for two reasons. First, as with the Younger Dryas transition, $\delta^{13}C_{wax}$ and δD_{wax} variations during the mid-Holocene track one another closely, and within the resolution of the data, there is no evidence that the vegetation changes more abruptly than hydrology. Furthermore, there is no clear evidence of any lag between the changes in $\delta^{13}C_{wax}$ and δD_{wax}. Thus, although the changes in $\delta^{13}C_{wax}$ appear to be large and occur rapidly, there is

nothing in the data to suggest a nonlinear vegetation response at that time. Furthermore, the deglacial transition differs from that of the mid-Holocene in that the $\delta^{13}C_{wax}$ values show very little variability leading up to the transition, even though there are significant variations in δD_{wax}. Thus, it appears that leading up to the deglaciation, some factor was preventing the vegetation from responding to changes in rainfall. We argue that this factor was fire, and that only once fire frequency was significantly reduced were woody plants able to establish themselves in the landscape. In contrast, both $\delta^{13}C_{wax}$ and δD_{wax} display significant, and often coherent, variations throughout the Holocene. Furthermore, it may be argued that a substantial component of the large, mid-Holocene vegetation shift examined here is the result of a shift towards higher $\delta^{13}C_{wax}$ values right before the vegetation shift.. The overall variability in Holocene $\delta^{13}C_{wax}$ values, the apparent synchrony of changes in $\delta^{13}C_{wax}$ and δD_{wax} during the mid Holocene C_3 plant expansion, and the lack of evidence for a lag between $\delta^{13}C_{wax}$ and δD_{wax} suggest the large Holocene vegetation response reflects a direct response to changes in precipitation, in contrast to the deglacial change. Instead, the magnitude of the mid-Holocene vegetation response, as well as the overall enhanced vegetation sensitivity, could reflect the effects of elevated CO_2, which allows for more rapid woody plant growth and recovery.

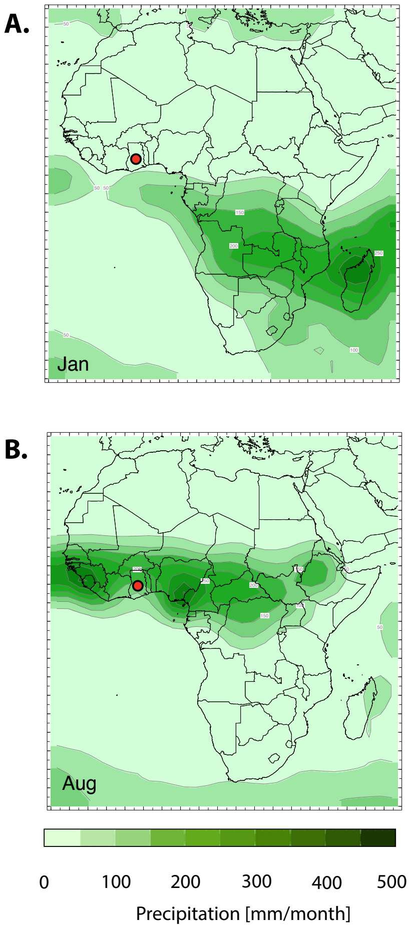

Figure S1. Seasonal variations in monthly precipitation over Africa based on average **A.** January and **B.** August precipitation over the period between 1981-2010 from the CAMS OPI 2.5° monthly averaged precipitation dataset[50]. Red circle indicates the location of Lake Bosumtwi. Figure generated with the Lamont-Doherty Earth Observatory International Research Institute data library (http://iridl.ldeo.columbia.edu).

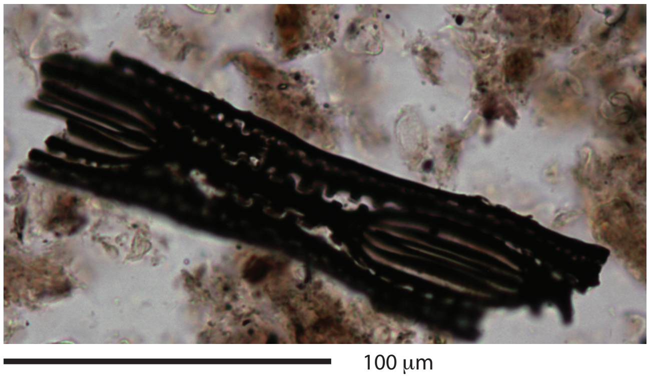

Figure S2. Transmitted light photograph of grass charcoal in a pollen slide from Lake Bosumtwi. Ghana. 400x magnification.

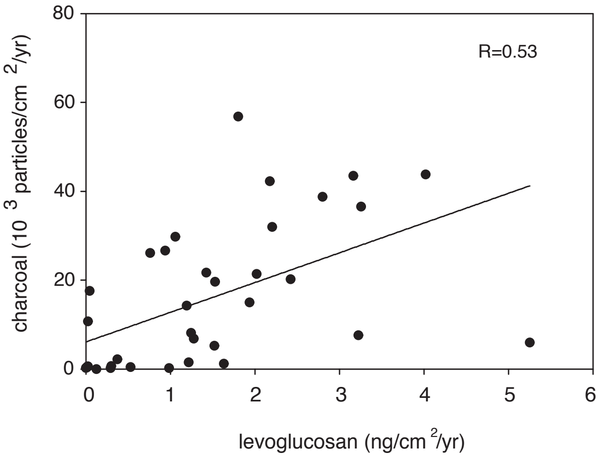

Figure S3. Correlation between charcoal counts and levoglucosan concentrations. Levoglucosan concentrations have been linearly interpolated to the same depths as the charcoal samples. All concentrations were normalized to time using the estimated number of years of deposition for the sample from either varve counts or the age model.

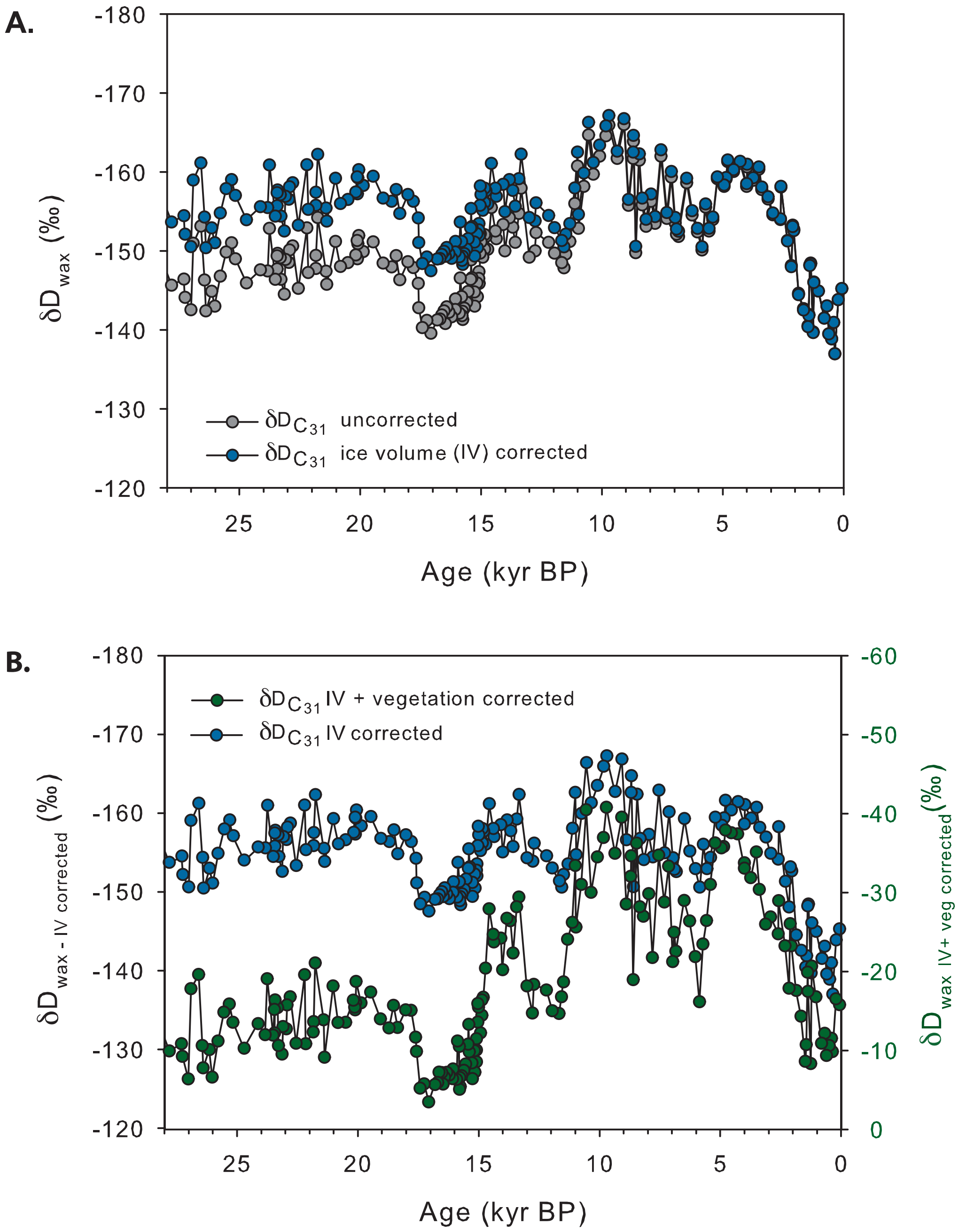

Figure S4. Corrections to plant wax hydrogen isotope data. A. Corrections for the influence of changing ice volume on the stable isotopic composition of the oceans. **B.** Corrections for the influence of plant functional type on the apparent fractionation during leaf wax synthesis

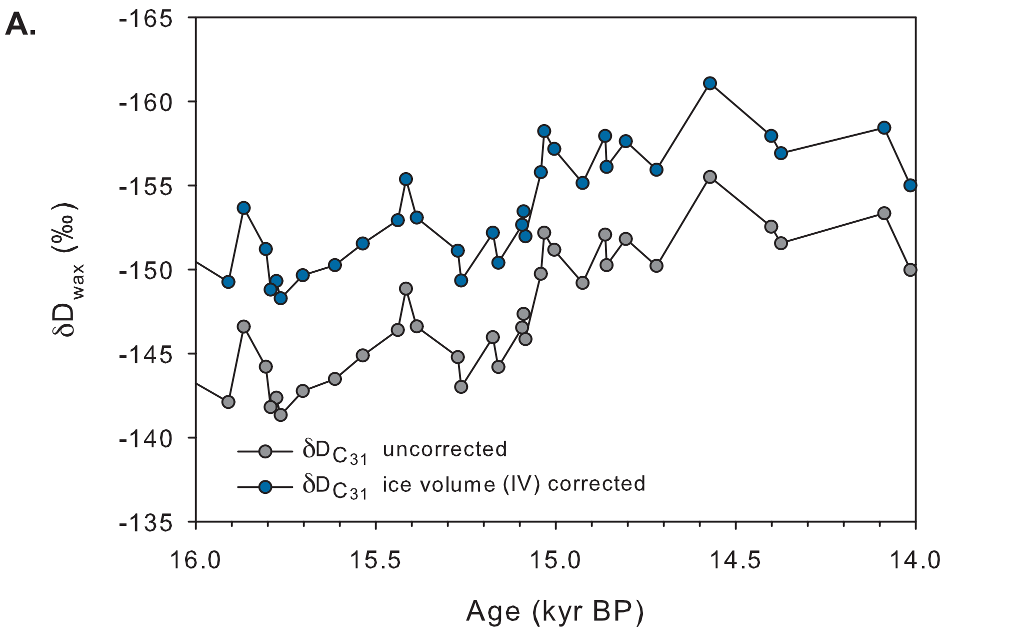

Figure S5. Expanded view of the corrections to plant wax hydrogen isotope data. Same as in Figure S4. The data highlight the fact that the corrections make here do not significantly alter the relative abruptness of the reconstructed hydrological changes.

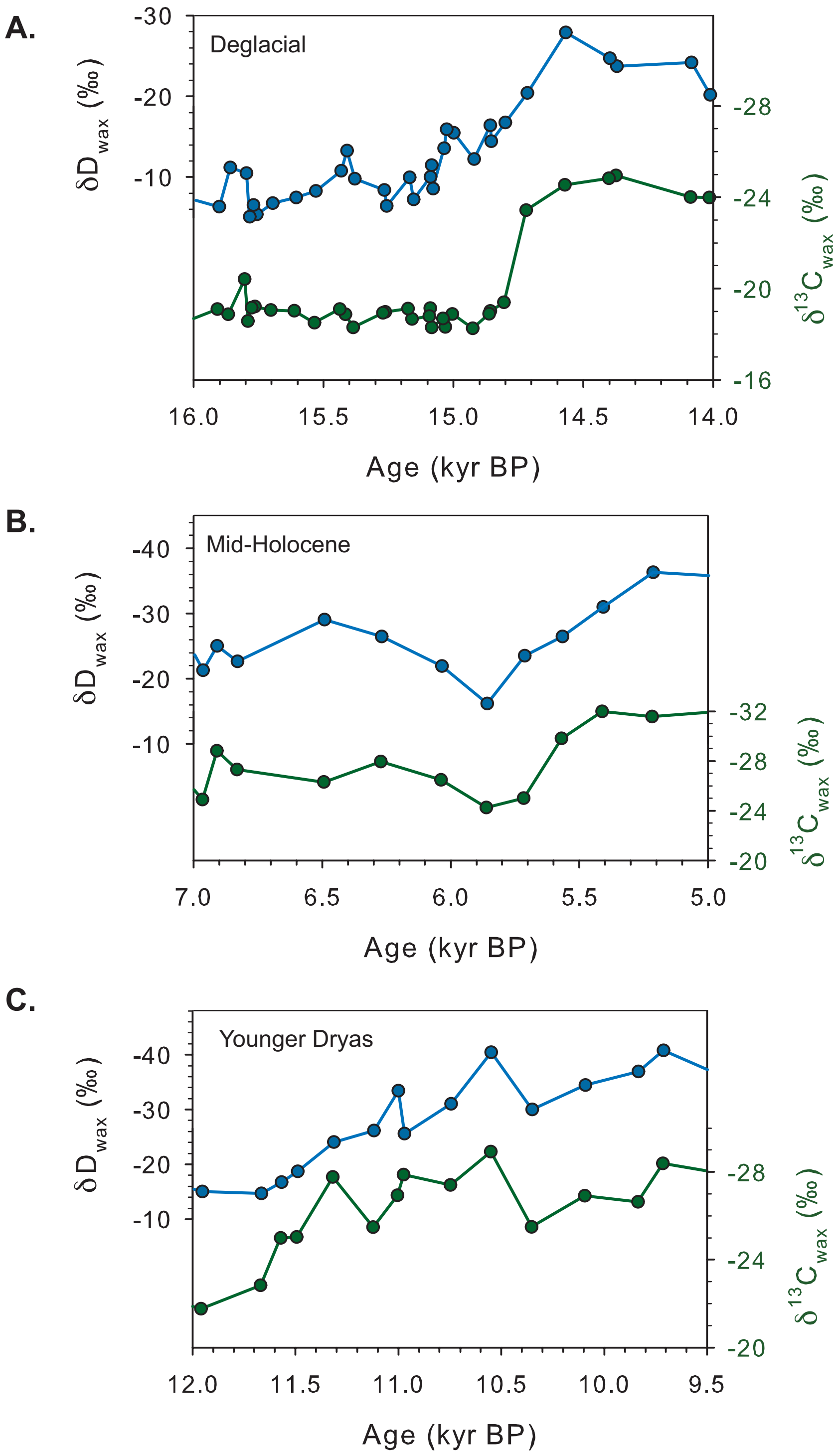

Figure S6. Comparison of the rate of change during the three most prominent vegetation shifts. Plotted are blowups of the plant wax hydrogen (blue) and carbon (green) isotope data for **A.** the deglacial (18-14 kyr BP), **B.** the mid-Holocene (7-5 kyr BP) and **C.** the Younger Dryas (12-9.5 kyr BP).

References

1 Shanahan, T. M. *et al.* Age models for long lacustrine sediment records using multiple dating approaches - An example from Lake Bosumtwi, Ghana. *Quaternary Geochronology* **15**, 47-60 (2013).

2 Shanahan, T. M. *et al.* The time-transgressive termination of the African Humid Period. *Nature Geoscience* **8**, 140-144 (2015).

3 Otto, A., Gondokusumo, R. & Simpson, M. J. Characterization and quantification of biomarkers from biomass burning at a recent wildfire site in Northern Alberta, Canada. *Applied Geochemistry* **21**, 166-183 (2006).

4 Elias, V., Simoneit, B., Cordeiro, R. & Turcq, B. Evaluating levoglucosan as an indicator of biomass burning in Carajas, Amazonia: A comparison to the charcoal record. *Geochimica et Cosmochimica Acta* **65**, 267-272 (2001).

5 Clark, J., Royall, P.D. Particle-Size Evidence for Souce Areas of Charcoal Accumulation in Late Holocene Sediments of Eastern North American Lakes. *Quaternary Research* **43**, 80-89 (1995).

6 Stockmarr, J. Tablets with spores used in absolute pollen analysis. *Pollen et Spore XIII*, 615 (1972).

7 Finsinger, W. & Tinner, W. Minimum count sums for charcoal concentration estimates in pollen slides: accuracy and potential errors. *The Holocene* **15**, 293-297 (2005).

8 Simoneit, B. R. Biomass burning—a review of organic tracers for smoke from incomplete combustion. *Applied Geochemistry* **17**, 129-162 (2002).

9 Simoneit, B. R. T. *et al.* Levoglucosan, a tracer for cellulose in biomass burning and atmospheric particles. *Atmospheric Environment* **3**, 173–182 (1999).

10 Clark, J. S. Fire and climate change during the last 750 yr in northwestern Minnesota. *Ecological Monographs* **60**, 135-159 (1990).

11 Gardner, J. J. & Whitlock, C. Charcoal accumulation following a recent fire in the Cascade Range, northwestern USA, and its relevance for fire-history studies. *The Holocene* **11**, 541-549 (2001).

12 Lynch, J. A., Clark, J. S. & Stocks, B. J. Charcoal production, dispersal, and deposition from the Fort Providence experimental fire: interpreting fire regimes from charcoal records in boreal forests. *Canadian Journal of Forest Research* **34**, 1642-1656 (2004).

13 Tinner, W. *et al.* Pollen and charcoal in lake sediments compared with historically documented forest fires in southern Switzerland since AD 1920. *The Holocene* **8**, 31-42 (1998).

14 Whitlock, C. & Millspaugh, S. H. Testing the assumptions of fire-history studies: an examination of modern charcoal accumulation in Yellowstone National Park, USA. *The Holocene* **6**, 7-15 (1996).

15 Kuo, L.-J., Herbert, B. E. & Louchouarn, P. Can levoglucosan be used to characterize and quantify char/charcoal black carbon in environmental media? *Organic Geochemistry* **39**, 1466-1478 (2008).

16 Kuo, L.-J., Louchouarn, P. & Herbert, B. E. Influence of combustion conditions on yields of solvent-extractable anhydrosugars and lignin phenols in chars: Implications for characterizations of biomass combustion residues. *Chemosphere* **85**, 797-805 (2011).

17 Leys, B., Brewer, S. C., McConaghy, S., Mueller, J. & McLauchlan, K. K. Fire history reconstruction in grassland ecosystems: amount of charcoal reflects local area burned. *Environmental Research Letters* **10**, 114009 (2015).

18 Still, C. J. & Powell, R. L. *Isoscapes: Understanding Movement, Pattern, and Process on Earth Through Isotope Mapping*. (Springer, 2010).

19 Collatz, G. J., Berry, J. A. & Clark, J. S. Effects of climate and atmospheric CO_2 partial pressure on the global distribution of C_4 grasses: present, past, and future. *Oecologia* **114**, 441–454 (1998).

20 Lloyd, J., Bird, M. I., Vellen, L., Miranda, A. C., Veenendaa,l E. M., Djagbletey, G., Miranda, H. S., Cook, G., Farquhar, G. D. Contributions of woody and herbaceous vegetation to tropical savanna ecosystem productivity: a quasi-global estimate. *Tree Physiology* **28**, 451–468 (2008).

21 Schefuß, E., Schouten, S. & Schneider, R. Climatic controls on central African hydrology during the past 20,000 years. *Nature* **437**, 1003-1006 (2005).

22 Castaneda, I. S., Werne, J. P., Johnson, T. C. Wet and arid phases in the southeast African tropics since the Last Glacial Maximum. *Geology* **35**, 823–826 (2007).

23 Collins , J. A., Schefuß, E., Heslop, D., Mulitza, S., Prange, M., Zabel, M., Tjallingii, R., Dokken, T. M., Huang, E., Mackensen, A., Schulz, M., Tian, J., Zarriess, M. and Wefer, G. Interhemispheric symmetry of the tropical African rainbelt over the past 23,000 years. *Nature Geoscience* **4**, 42–45 (2011).

24 Diefendorf, A. F., Mueller, K. E., Wing, S. L., Koch, P. L. & Freeman, K. H. Global patterns in leaf 13C discrimination and implications for studies of past and future climate. *Proceedings of the National Academy of Sciences* **107**, 5738-5743, doi:10.1073/pnas.0910513107 (2010).

25 Garcin, Y. *et al.* Reconstructing C_3 and C_4 vegetation cover using n-alkane carbon isotope ratios in recent lake sediments from Cameroon, Western Central Africa. *Geochimica et Cosmochimica Acta* **142**, 482-500 (2014).

26 Diefendorf, A. F., Mueller, K. E., Wing, S. L., Koch, P. L., & Freeman, K. H. Global patterns in leaf ^{13}C discriminationand implications for studies of past and future climate. *Proceedings of the National Academy of Sciences* **107**, 5738–5743 (2010).

27 Farquhar, G., O'Leary, M. & Berry, J. On the Relationship Between Carbon Isotope Discrimination and the Intercellular Carbon Dioxide Concentration in Leaves. *Functional Plant Biology* **9**, 121-137 (1982).

28 Kaplan, J. O., Prentice, C., Buchmann, N . The stable carbon isotope composition of the terrestrial biosphere: Modeling at scales from the leaf to the globe. *Global Biogeochem Cycles* **16**, 1060 (2002).

29 Kohn, M. J. Carbon isotope compositions of terrestrial C_3 plants as indicators of (paleo)ecology and (paleo)climate. *Proceedings of the National Academy of Sciences* **107**, 19691–19695 (2010).

30 van der Merwe, N. J. & Medina, E. The canopy effect, carbon isotope ratios and foodwebs in Amazonia. *Journal of Archaeological Science* **18**, 249–259 (1991).

31 Maley, J. in *Paleoclimatology and Paleometeorology: Modern and Past Patterns of Global Atmospheric Circulation* (eds M. Leinen & M. Sarnthein) 585-616 (Kluwer Academic Publishers, 1989).
32 Njitchoua, R. *et al.* Variations of the stable isotopic compositions of rainfall events from the Cameroon rain forest, Central Africa. *Journal of Hydrology* **223**, 17-26 (1999).
33 Sachse, D. *et al.* Molecular Paleohydrology: Interpreting the Hydrogen-Isotopic Composition of Lipid Biomarkers from Photosynthesizing Organisms. *Annu. Rev. Earth Planet. Sci.* **40**, 221-249 (2012).
34 Tierney, J. E., Russell, J. M., Damste, J. S. S., Huang, Y. & Verschuren, D. Late Quaternary behavior of the East African monsoon and the importance of the Congo Air Boundary. *Quaternary Science Reviews* **30**, 798-807, doi:10.1016/j.quascirev.2011.01.017 (2011).
35 Schrag, D. P., Hampt, G. & Murray, D. W. Pore fluid constraints on the temperature and oxygen isotopic composition of the glacial ocean. *Science* **272**, 1930-1932 (1996).
36 Shackleton, N. J. The 100,000-year ice-age cycle identified and found to lag temperature, carbon dioxide, and orbital eccentricity. *Science* **289**, 1897-1902 (2000).
37 Vimeux, F. *et al.* A 420,000 year deuterium excess record from East Antarctica: Information on past changes in the origin of precipitation at Vostok. *Journal of Geophysical Research-Atmospheres* **106**, 31863-31873 (2001).
38 Lisiecki, L. E. & Raymo, M. E. A Pliocene-Pleistocene stack of 57 globally distributed benthic delta O-18 records. *Paleoceanography* **20,** PA1003 (2005).
39 Garcin, Y. *et al.* Hydrogen isotope ratios of lacustrine sedimentary n-alkanes as proxies of tropical African hydrology: Insights from a calibration transect across Cameroon. *Geochimica et Cosmochimica Acta* **79**, 106-126 (2012).
40 Bi, X., Sheng, G., Liu, X., Li, C., Fu, J. . Molecular and carbon and hydrogen isotopic composition of n-alkanes in plant leaf waxes. *Organic Geochemistry* **36**, 1405-1417 (2005).
41 Chikaraishi , Y., Naraoka, H. Compound-specific δD– δ^{13}C analyses of n-alkanes extracted from terrestrial and aquatic plants. *Phytochemistry* **63**, 361–371 (2003).
42 Hou, J., D'Andrea, W. J., MacDonald, D., Huang, Y. . Hydrogen isotopic variability in leaf waxes among terrestrial and aquatic plants around Blood Pond, Massachusetts (USA). *Organic Geochemistry* **38**, 977–984 (2007).
43 Pedentchouk, N., Sumner, W., Tipple B., Pagani ,M. δ^{13}C and δD compositions of n-alkanes from modern angiosperms and conifers: an experimental set up in central Washington State, USA. *Organic Geochemistry* **39**, 1066-1071 (2008).
44 McInerney, F. A., Helliker, B. R., Freeman, K. H. Hydrogen isotope ratios of leaf wax n-alkanes in grasses are insensitive to transpiration. *Geochimica et Cosmochimica Acta* **75**, 541-554 (2011).
45 Liu, W. G., Yang, H. . Multiple controls for the variability of hydrogen isotopic compositions in higher plant n-alkanes from modern ecosystems. *Global Change Biology* **14**, 2166–2177 (2008).

46 Liu, W. G., Yang, H., Li, L. Hydrogen isotopic compositions of n-alkanes from terrestrial plants correlate with their ecological life forms. *Oecologia* **150**, 330–338 (2006).

47 Collins, J. A. *et al.* Estimating the hydrogen isotopic composition of past precipitation using leaf-waxes from western Africa. *Quaternary Science Reviews* **65**, 88-101 (2013).

48 Polissar, P. J., Freeman, K. H. . Effects of aridity and vegetation on plant-wax δD in modern lake sediments. *Geochimica et Cosmochimica Acta* **74**, 5785-5797. (2010).

49 Smith, F. A., Freeman, K. H. . Influence of physiology and climate on δD of leaf wax n-alkanes from C3 and C4 grasses. *Geochimica et Cosmochimica Acta* **70**, 1172-1187 (2006).

50 Janowiak, J. E. & Xie, P. P. CAMS_OPI: A Global Satellite-Rain Gauge Merged Product for Real-Time Precipitation Monitoring Applications. *J. Clim.* **12**, 3335-3342 (1999).
